# Effects of Rehabilitative Exercise and Neuromuscular Electrical Stimulation on Muscle Morphology and Dynamic Balance in Individuals with Chronic Ankle Instability

**DOI:** 10.3390/medicina60071187

**Published:** 2024-07-22

**Authors:** Sujin Choi, Hyung-pil Jun

**Affiliations:** Department of Physical Education, Dong-A University, Busan 49315, Republic of Korea; sjchoi856793@gmail.com

**Keywords:** chronic ankle instability, muscle morphology, dynamic balance, rehabilitative exercise, neuromuscular electrical stimulation

## Abstract

*Background and Objectives:* Muscle atrophy caused by chronic ankle instability (CAI) can incur muscle weakness, altered movement patterns, and increased risk of injury. Previous studies have investigated the effects of rehabilitative exercises and neuromuscular electrical stimulation (NMES) on characteristics in CAI individuals, but few studies have examined their effects on foot and ankle muscle morphology. This study aimed to determine the effects of rehabilitative exercises and NMES on muscle morphology and dynamic balance in individuals with CAI. *Materials and Methods:* Participants with CAI (*n* = 47) were randomly divided into control (CG), rehabilitative exercise (REG), NMES (NG), and rehabilitative exercise and NMES combined (RNG) groups. The six-week intervention program consisting of rehabilitative exercises and NMES was applied to groups excluding CG. Muscle morphology and dynamic balance were evaluated using a portable wireless diagnostic ultrasound device and dynamic balance tests. For statistical analysis, an effect size with 95% confidence interval was calculated to assess mean differences according to intervention. *Results:* After six weeks, significant increases in morphology and dynamic balance were observed for all muscles except flexor hallucis longus (*p* > 0.05) in the intervention groups except for CG. However, no significant changes were observed in the CG (*p* > 0.05). *Conclusions:* These findings suggest that intervention programs may help prevent muscle atrophy and improve balance in CAI individuals.

## 1. Introduction

Lateral ankle sprain (LAS), which occurs in approximately 2 million cases each year in the United States [1], is one of the most common musculoskeletal injuries occurring in the lower extremities [2], with a high incidence in physically active populations such as athletes and military personnel [3].

Approximately 40% of individuals who experience an initial ankle sprain may develop chronic ankle instability (CAI) with persistent residual symptoms such as pain, recurrent sprains, and frequent episodes of the ankle giving way [2,4]. Two potential contributors to CAI are mechanical instability (lateral ligament laxity after a sprain) and functional instability (instability related to decreased proprioception or neuromuscular control) [2]. Moreover, individuals with CAI exhibit features such as muscle atrophy [5], muscle weakness [5], and reduced dynamic balance [6] when compared to those without CAI. The ankle joint complex consists of the lower leg and foot, and it plays an important role in forming the kinetic linkage that allows for the lower limb to interact with the ground [7]. If CAI is left untreated, it can have an altered movement patterns [8] and cause secondary injuries such as post-traumatic osteoarthritis [3], so appropriate management is necessary.

Most people tend to choose the various interventions such as preventative taping [9], rehabilitative exercises [10], and electrical stimulation [11] to improve CAI. Rehabilitative exercises and neuromuscular electrical stimulation (NMES) are particularly recognized as key roles in the recovery of ankle function [10,11,12]. Among the various rehabilitation exercises, balance training is an effective method of addressing imbalances that can potentially lead to ankle sprains and subsequent recurrences [13]. In a previous study, it was found that the balance deficits in CAI were improved through six weeks of balance rehabilitation exercises [10]. This also led to enhancements in functional performance [14,15] and perceived stability [16]. Strengthening muscle strength is essential because the strength of the gastrocnemius and soleus muscles, which are the muscles of both joints that affect the stability of the ankle joint, also affect dynamic and static balance [17,18]. Calf raise exercise is a representative method to strengthen the gastrocnemius and soleus muscles [19]. Several studies have demonstrated a positive outcome in terms of increased muscle morphology when performing this exercise [20,21,22]. When these exercises were conducted on varying types of support surfaces, it was found that exercising on an unstable support surface significantly increased the thickness of the gastrocnemius muscle compared to a stable support surface [21]. Furthermore, a significant improvement in gastrocnemius thickness was observed when eccentric calf raises were performed for six weeks, in comparison to normal calf raises [22]. Thus, the calf raise exercise is considered an effective method for the prevent or rehabilitate of ankle injuries.

Additionally, NMES is used to strengthen muscles to prevent loss of both muscle volume and strength [23]. It is designed to induce depolarization of the motor endplate, which is caused by artificially provoking muscle contractions via the application of electrical stimulation through the skin to stimulate motor nerves [23]. Recent studies have shown that NMES has a significant positive effect on muscle function and morphology. When individuals with anterior cruciate ligament reconstruction, knee osteoarthritis, or postoperative knee instability were treated with NMES, there was significant improvement in quadriceps pain, strength, and morphology [24,25,26]. Furthermore, an enhancement in the muscle morphology of the tibialis anterior and gastrocnemius muscles was observed in children with cerebral palsy who underwent a combination of NMES and exercises, compared to those who only received exercises [27]. As a result, rehabilitative exercises like balance training, strength training, and application of NMES have proven to be effective strategies for managing pain [24], enhancing dynamic balance [14,15], and improving morphology [27]. Therefore, it is important to apply these interventions for the prevention or treatment of ankle instability.

While numerous studies have investigated the impact of rehabilitative exercise and NMES, there is a lack of research investigating the effect of interventions on muscle morphology and dynamic balance in CAI. Hence, this study aims to determine the influence of rehabilitative exercises and the use of NMES on the morphology of foot and ankle muscles, as well as the dynamic balance in individuals with CAI. This study hypothesizes that the intervention group will show improvements in muscle morphology and dynamic balance compared to before the application.

## 2. Materials and Methods

### 2.1. Study Design

This study was a randomized controlled trial. The participants who met the inclusion criteria of CAI were randomly assigned to the intervention groups (rehabilitative exercise or NMES or combination) or the control group.

### 2.2. Participants

A total of 47 physically active individuals with CAI voluntarily participated in this study (Table 1). Physically active individuals were recruited from university according to American College of Sports Medicine (ACSM) guidelines [28]. The criteria corresponding to CAI were adapted from the previous study [29] as follows: (1) a history of at least 1 significant LAS, (2) a Cumberland Ankle Instability Tool (CAIT) questionnaire score ≤ 24, and (3) an Identification of Functional Ankle Instability (IdFAI) questionnaire score ≥ 11. Exclusion criteria included the followings: (1) A history of lower extremity surgery or fracture within the last 3 months, (2) an acute injury to the lower extremity in the past 6 weeks, (3) ultrasound cannot be observed for reasons such as swelling or bruising, (4) a musculoskeletal lesion of the muscles of the foot and/or ankle muscle such as arthritis, (5) administering pharmacotherapy such as muscle relaxants or chemotherapy, and (6) had difficulty performing dynamic balance tests. They were randomly allocated into four groups: a control group (CG), a rehabilitative exercise group (REG), a neuromuscular electrical stimulation group (NG), and a group that combined rehabilitative exercise and neuromuscular electrical stimulation (RNG). A six-week protocol of rehabilitative exercise and NMES was implemented for all groups, except the CG.

The calculation of the necessary sample size was conducted through a power analysis. This analysis considered a minimum statistical power of 0.8 (1-β), an α-level of 0.5, 4 testing conditions, and a moderate effect size of 0.25, referring to studies with similarities to the current study design [30]. The outcome of this analysis suggested that a total of 48 participants would be needed. This computation was carried out using the G-power Power Analysis software package (ver. 3.1.9.7) from Universitat Kiel, Germany.

### 2.3. Procedure

**Figure 1:**
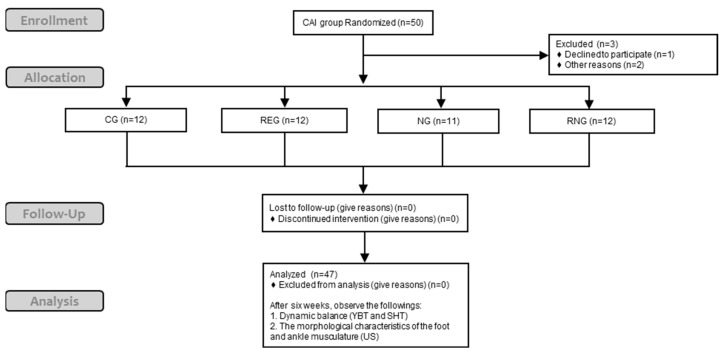
Consort flow chart.

Fifty individuals with CAI were screened, 1 of them declined to participate, and 2 dropped out due to personal reasons, resulting in a total of 47 participants participating in this study. A total of 47 participants with CAI who participated in this study were randomly assigned to four groups: control group (CG), rehabilitative exercise group (RG), neuromuscular electrical stimulation group (NG), or mixed group (RNG). Muscle morphology and dynamic balance were measured for all participants before applying the intervention. Excluding the CG, the three groups received under supervision intervention three times a week for a total of six weeks. After six weeks, muscle morphology and dynamic balance were retaken (Figure 1).

### 2.4. Instruments

#### 2.4.1. Portable Wireless Diagnostic Ultrasound Device

A portable wireless diagnostic ultrasound device (SONON 300L, Healcerion Inc., Seoul, Republic of Korea) and a portable tablet (iPad, Apple Inc., Cupertino, CA, USA) were used in this experiment to measure the morphology [cross-sectional area (CSA) and muscle thickness] of the foot and ankle muscles of participants with CAI. To obtain accurate muscle morphology images, hair was removed from the measurement point. Ultrasound equipment [B mode (Gain: 53%; DR: 54 dB; TGC: 48%/filter → Frame average: 3; SRI: 4; gray map: F)] was set following the guidelines of a prior research study [31]. Measurements of muscle morphology were performed by a single examiner to establish intra-rater reliability.

Previous studies were referred to observe the morphology of the deep muscles of the lower leg [32,33]. First, leg hair must be removed from the measurement area to obtain a clear image before measuring the morphology of the foot and ankle muscles. Then, the examiner instructed each participant to assume a supine position on the examination table to scan the flexor digitorum longus (FDL) and flexor hallucis longus (FHL). To measure CSA and muscle thickness, the probe was placed perpendicular and parallel to the muscle belly, respectively. At the point halfway between the medial plateau and the inferior border of the medial malleolus, FDL muscle was observed. The reliability of both CSA and MT of FDL was excellent, with ICC_3,1_ of 0.92 and 0.93, respectively. The morphology of the FHL was observed at a position slightly backward to the measurement point of the FDL. The reliability of both CSA and MT of the FHL was excellent with ICC_3,1_ of 0.97 and 0.99, respectively.

Each participant was instructed to assume a prone position, with their feet out of the examination table. The gastrocnemius (GA) was divided into lateral (GA-L), and medial (GA-M) heads based on the mid-line. Similar to the morphology measure methods of the FDL and FHL muscles, the probe was placed perpendicular and parallel to the muscle belly to measure their CSA and muscle thickness, respectively. Both CSA and MT of GA-M had excellent reliability, with ICC_3,1_ of 0.93 and 0.95, respectively. Both the CSA and MT of GA-L also had excellent reliability, with an ICC_3,1_ of 0.93 and 0.96, respectively. The morphology of the soleus (SOL) was observed at the halfway point of the calf muscle. The reliability of both the CSA and MT of SOL was excellent with an ICC_3,1_ of 0.95 and 0.96, respectively. The morphology of the superficial muscles of the lower leg was observed with reference to the protocol used in similar studies [34,35].

#### 2.4.2. Dynamic Balance Test

**Figure 2:**
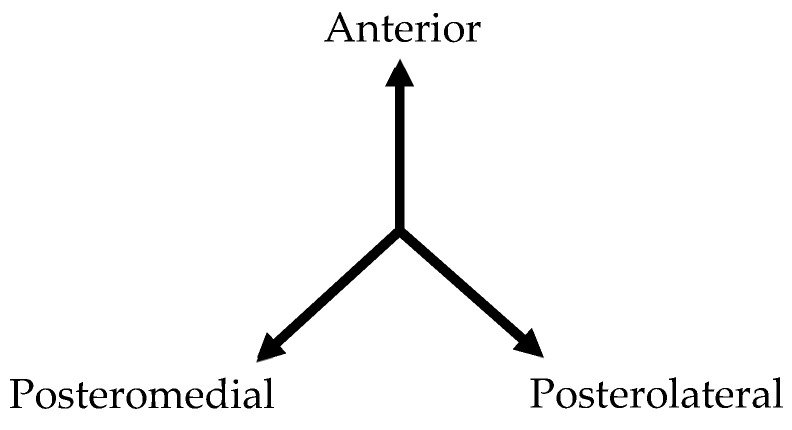
Three directions of Y-Balance test for the right limb.

This research utilized the Y-Balance kit^TM^ (Y-Balance Kit, Functional Movement System^TM^, Danville, VA, USA) to evaluate dynamic balance. The Y-Balance test (YBT) was conducted six times in each direction, followed by three times in the sequence of anterior (ANT), posteromedial (PM), and posterolateral (PL) (Figure 2). An examiner guided the participants to position themselves on the stance plate and use their toes to extend the reach indicator as far as they could in each direction [36]. By the YBT protocol, a trial was invalidated and redone under the following conditions: (1) inability to hold the posture on the stance plate (i.e., the reaching foot touched the floor), (2) failure to keep contact between the reaching indicator and the reaching foot in the target area (i.e., the reach indicator was kicked), (3) use of reach indicators to maintain a stable posture, (4) failure to keep the sole of the stance foot in contact with the stance plate (i.e., the heel was lifted), or (5) inability to return the reaching foot to the starting position (i.e., unsteadiness) [37]. The ICC for intra-rater reliability varied from 0.85 to 0.91, while the ICC for inter-rater reliability ranged from 0.99 to 1.00. The composite reach score reliability was 0.91 for intra-rater and 0.99 for inter-rater reliability [37].

**Figure 3:**
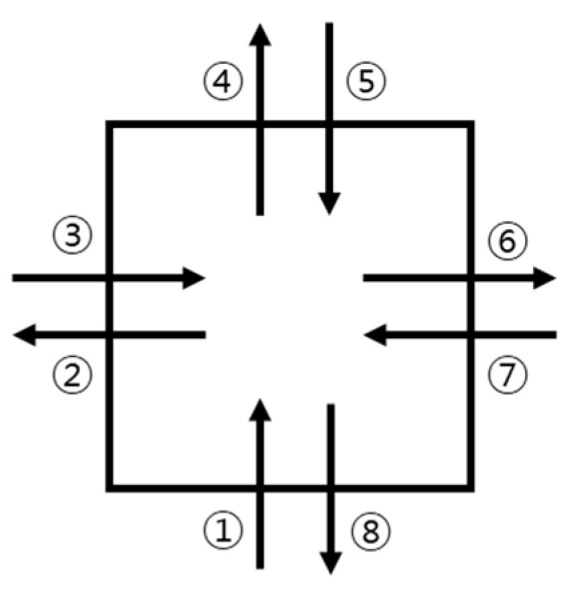
Order of square hop test of the right limb (clockwise). Numbers explain the order of steps.

For the square hop test (SHT), a square of 40 cm on each side was utilized following the testing protocol [6]. Participants were directed by an examiner to position themselves outside the square, ready themselves, and then enter and exit the square five times as rapidly as they could. Each leap over the boundary line of the square and return to the starting position was considered one repetition. The right limb was used in a clockwise direction, while the left limb was used in a counterclockwise direction (Figure 3). A trial was deemed invalid and redone under the following circumstances: (1) the other foot contacted the ground, (2) the direction was incorrect, (3) there were stopwatch-related errors (i.e., it was turned off), and (4) the participant did not fully cross the square line (i.e., they stepped on the square line). The reliability of this test was good, with an ICC_2,1_ of 0.90 (SEM: 1.40 s; MDC95: 3.88 s) [6].

### 2.5. Interventions

#### 2.5.1. Rehabilitative Exercise

In this research, a six-week rehabilitative exercise was implemented, which consisted of heel raise and wobble board exercises (Table 2). The exercises were modified versions of those found in previous studies [21,22]. The participant positioned their affected foot on a balance pad (Thera-band^®^, Akron, OH, USA), with a slight bend in the knee, while the other foot remained on the ground. Both heels were then lifted as high as possible. The participant lifted their non-affected leg and balanced on the pad with the affected foot for three seconds. At this point, the heel of the standing foot was lowered while creating eccentric loading. Each participant completed 15 repetitions per set, with three training sessions each week. An additional rehabilitative exercise involved a wobble board exercise, aimed at enhancing both static and dynamic balance for those with ankle instability. This exercise utilized a wobble board (TheraBand^®^ Stability Trainers, The Hygenic Corporation, Akron, OH, USA) [15]. Participants were guided to stand on one leg on the wobble board and perform movements in both clockwise and counterclockwise directions, with the option to stabilize themselves by touching the wall with their fingertips [15]. Prior to starting, no initial direction was specified, and participants were instructed to switch directions every 10 s for a total duration of 40 s. This exercise was also performed in three training sessions each week [15].

#### 2.5.2. Neuromuscular Electrical Stimulation (NMES)

In this study, the EMS 7500 device (Balego & Associates, Saint Paul, MN, USA) was utilized. Over six weeks, the NMES protocol was applied three times weekly, with sessions held once daily, each lasting 30 min. Participants were instructed to maintain clean, alcohol-dried skin and remove hair from the affected leg. The parameters, guided by systematic reviews [26], were as follows: a frequency of 30 Hz, pulse width of 300 µs, with 10 s on and 30 s off cycles and the intensity was set to a level acceptable to each participant. Electrodes were attached, with two placed on the GA and two on the lower third aspects of the medial lower leg, specifically targeting the pathway of the FDL.

### 2.6. Data Processing

**Figure 4:**
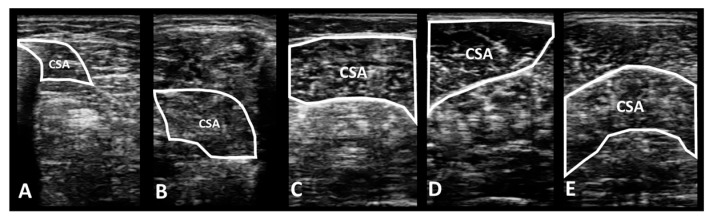
Measurement of muscle cross-sectional area using ImageJ software. CSA: cross-sectional area. (**A**) CSA of flexor digitorum longus; (**B**) CSA of flexor hallucis longus; (**C**) CSA of gastrocnemius medial head; (**D**) CSA of gastrocnemius lateral head; (**E**) CSA of soleus.

**Figure 5:**
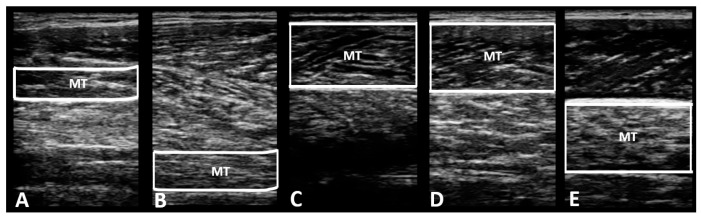
Measurement of muscle thickness using ImageJ software. MT: muscle thickness. (**A**) MT of flexor digitorum longus; (**B**) MT of flexor hallucis longus; (**C**) MT of gastrocnemius medial head; (**D**) MT of gastrocnemius lateral head; (**E**) MT of soleus.

The collected images of muscle morphology were processed using ImageJ software https://imagej.net/ij/download.html (National Institute for Health, Bethesda, MD, USA), and the images of each muscle were measured three times to derive the average value. The average values of CSA (Figure 4) and muscle thickness (Figure 5) for each muscle were used for data analysis.

Given the correlation between reach and limb length, the reach distance was normalized relative to the participant’s limb length [38]. The normalized reach distance was computed by dividing the average of three reaches by the limb length and then multiplying by 100. A composite score was determined by dividing the sum of the three directional reaches by three times the limb length, followed by multiplication by 100.

$$Absolute\;reach\;distance\;(cm)\;=\;\frac{Trial\;1\;+\;Trial\;2\;+\;Trial\;3}{3}$$

$$Relative\;\left(normalized\right)\;reach\;distance\;(\%)\;=\;\frac{Absolute\;reach\;distance}{Leg\;length}\;\times \;100$$

$$Composite\;score\;(\%)\;=\;\frac{Sum\;of\;the\;3\;reach\;directions}{3\;\times \;Leg\;length}\times \;100$$

An examiner used a stopwatch to measure the time required to complete five repetitions, recording the time to the nearest hundredth of a second.

### 2.7. Statistical Analysis

The SPSS version 26.0 (IBM SPSS Inc., Chicago, IL, USA) was used for statistical analysis, and all data were presented as mean ± standard deviation (SD). The Shapiro–Wilk test was used to examine the normality of data distribution and confirmed that the data followed a normal distribution. In this study, the effect size was calculated to compare the mean difference according to the application of the intervention within the group, which was presented as Cohen’s *d* with 95% confidence interval. To calculate Cohen’s *d* within the group, the difference between post-intervention and baseline was divided by the SD of baseline. Effect size *d* was interpreted as follows: *d* < 0.2, very small; 0.2 ≤ *d* < 0.5, small; 0.5 ≤ *d* < 0.8, moderate; 0.8 ≤ *d* < 1.2, large; 1.2 ≤ *d*, very large. If the 95% confidence interval includes “0”, it means it is not statistically significant. For all analysis, the significance level was set a priori at α = 0.05.

## 3. Results

### 3.1. Changes in the Morphological Characteristics of the Foot and Ankle Muscle According to Each Intervention Methods

Table 3 is showed the morphological changes in the foot and ankle muscles between the CAI groups.

#### 3.1.1. Deep Muscles in the Lower Leg

- Flexor digitorum longus

In this study, significant differences were found in the muscle morphology of the FDL in groups excluding the CG. The REG significantly improved the CSA (pre: 0.86 ± 0.10; post: 1.21 ± 0.21) and muscle thickness (pre: 0.83 ± 0.07; post: 0.99 ± 0.13) for FDL after intervention, which showed a very large effect size (*d* = 2.07 [1.08 to 3.06]; *d* = 1.54 [0.63 to 2.45]). The results showed that not only the CSA (pre: 0.87 ± 0.07; post: 1.19 ± 0.15) of the NG but also muscle thickness (pre: 0.84 ± 0.08; post: 0.98 ± 0.13) was significantly increased, and the effect size was very large (*d* = 2.67 [1.52 to 3.82]; *d* = 1.21 [0.30 to 2.12]), respectively. In addition, the RNG showed similar results to other intervention groups. The CSA (pre: 0.86 ± 0.08; post: 1.25 ± 0.27; *d* = 2.02 [1.04 to 3.00]) and muscle thickness (pre: 0.81 ± 0.07; post: 1.04 ± 0.12; *d* = 2.43 [1.38 to 3.48]) were significantly improved compared to before the intervention, and a very large effect size was observed. On the other hand, there was no significant change in CSA (pre: 0.84 ± 0.17; post: 0.82 ± 0.18) and muscle thickness (pre: 0.80 ± 0.09; post: 0.79 ± 0.09) of CG, and the effect size was very small (*d* = −0.16 [−0.96 to 0.64]; *d* = −0.14 [−0.94 to 0.66]).

- Flexor hallucis longus

No significant morphological changes were observed in the FHL muscle in any group over the past six weeks. Compared to six weeks ago, there was no change in either the CSA (pre: 4.95 ± 0.32; post: 4.98 ± 0.30) or thickness (pre: 1.36 ± 0.08; post: 1.37 ± 0.09) of the CG, and the effect size was too small to be statistically significant, respectively (*d* = 0.08 [−0.72 to 0.88]; *d* = 0.14 [−0.66 to 0.94]). Furthermore, no significant changes were observed in the group that applied the intervention. In the REG, there were no changes in either the CSA (pre: 4.97 ± 0.28; post: 4.97 ± 0.27) or thickness (pre: 1.36 ± 0.05; post: 1.37 ± 0.06) of the FHL, indicating a very small effect size, respectively (*d* = 0.02 [−0.78 to 0.82]; *d* = 0.15 [−0.66 to 0.95]). There were no significant differences in either the CSA or thickness of the NG and RNG groups, regardless of the intervention method. The effect size for the CSA (pre: 4.96 ± 0.41; post: 4.97 ± 0.37; *d* = 0.03 [−0.81 to 0.87]) and thickness (pre: 1.36 ± 0.06; post: 1.37 ± 0.04; *d* = 0.24 [−0.60 to 1.08]) of the FHL in the NG was very small and statistically insignificant. Similarly, the effect size for the CSA (pre: 4.97 ± 0.26; post: 4.98 ± 0.27; *d* = 0.04 [−0.76 to 0.84]) and thickness (pre: 1.36 ± 0.07; post: 1.37 ± 0.09; *d* = 0.10 [−0.70 to 0.90]) of the FHL in the RNG was also very small and statistically insignificant.

#### 3.1.2. Superficial Muscles in the Lower Leg

- Gastrocnemius medial head

In the case of the GA-M muscle, the CG did not show any significant alterations in either CSA (pre: 6.81 ± 1.13; post: 6.80 ± 0.94; *d* = 0.00 [−0.80 to 0.80]) or muscle thickness (pre: 1.65 ± 0.12; post: 1.66 ± 0.07; *d* = 0.06 [−0.74 to 0.86]) before and after the intervention, resulting in negligible effect sizes for both parameters. Conversely, the REG, NG, and RNG all demonstrated significant increases in both CSA and thickness following the intervention. In the REG, there were significant changes in both CSA (pre: 6.78 ± 0.57; post: 7.47 ± 0.83; *d* = 0.96 [0.12 to 1.81]) and thickness (pre: 1.64 ± 0.12; post: 1.90 ± 0.17; *d* = 1.83 [0.88 to 2.78]), as shown by the large to very large effect sizes. Similarly, the NG demonstrated significant increases in CSA (pre: 6.77 ± 0.71; post: 7.37 ± 0.65; *d* = 0.87 [0.03 to 1.70]) and thickness (pre: 1.64 ± 0.11; post: 1.82 ± 0.15; *d* = 1.37 [0.45 to 2.30]), with large to very large effect sizes. Furthermore, the RNG showed significant enhancements in both CSA (pre: 6.78 ± 1.05; post: 7.57 ± 0.76; *d* = 0.87 [0.03 to 1.70]) and thickness (pre: 1.64 ± 0.23; post: 1.96 ± 0.21; *d* = 1.48 [0.58 to 2.38]), and the effect sizes were large to very large.

- Gastrocnemius lateral head

The CG did not exhibit a significant change in the CSA (pre: 5.08 ± 0.59; post: 5.11 ± 0.50; *d* = 0.05 [−0.75 to 0.86]) and muscle thickness (pre: 1.35 ± 0.17; post: 1.36 ± 0.12; *d* = 0.09 [−0.71 to 0.89]) of the GA-L muscle over time, with very small effect sizes observed for both parameters. In contrast, the REG, NG, and RNG demonstrated significant increases in CSA and thickness after each intervention. REG showed a significant increase in CSA (pre: 5.02 ± 0.92; post: 6.16 ± 1.39; *d* = 0.97 [0.12 to 1.81]) and thickness (pre: 1.36 ± 0.25; post: 1.63 ± 0.24; *d* = 1.12 [0.26 to 1.98]) with large effect sizes. The NG exhibited significant increases in CSA (pre: 5.06 ± 0.56; post: 5.97 ± 0.74; *d* = 1.39 [0.12 to 1.81]) and thickness (pre: 1.37 ± 0.21; post: 1.59 ± 0.12; *d* = 1.36 [0.43 to 2.28]) with very large effect sizes. Similarly, RNG displayed significant increases in CSA (pre: 5.03 ± 0.57; post: 6.32 ± 0.45; *d* = 2.50 [1.43 to 3.57]) and thickness (pre: 1.36 ± 0.11; post: 1.66 ± 0.15; *d* = 2.26 [1.24 to 3.28]) with very large effect sizes.

- Soleus

In the morphology of SOL, there were significant changes in CSA and muscle thickness in the intervention groups excluding the CG. Compared to six weeks before, there was no change in the CSA (pre: 6.06 ± 0.60; post: 6.09 ± 0.36; *d* = 0.07 [−0.74 to 0.87]) and muscle thickness (pre: 1.54 ± 0.18; post: 1.54 ± 0.17; *d* = 0.02 [−0.78 to 0.82]) of the CG, and the effect size was not statistically significant. In contrast, the REG showed significant differences compared with pre-intervention in both CSA (pre: 5.82 ± 0.60; post: 6.95 ± 0.56; *d* = 1.59 [0.67 to 2.51]) and thickness (pre: 1.52 ±0.14; post: 1.80 ± 0.15; *d* = 1.94 [0.97 to 2.91]), with very large effect sizes found. Similarly, the NG showed a significant increase in both CSA (pre: 5.90 ± 0.82; post: 6.68 ± 0.80) and thickness (pre: 1.53 ± 0.13; post: 1.76 ± 0.16), with a large (CSA: *d* = 0.96 [0.08 to 1.85]) to very large (thickness: *d* = 1.59 [0.64 to 2.55]) effect size after applying the intervention. Like those of the REG and NG, there was also a statistically significant difference in both CSA and thickness in the RNG between before/after intervention. The effect size was very large (CSA: *d* = 2.26 [1.23 to 3.28]; thickness: *d* = 1.64 [0.72 to 2.57]), which is statistically significant.

### 3.2. Changes in Dynamic Balance on Y-Balance Test and Square Hop Test between Groups According to Each Intervention Method

#### 3.2.1. Y-Balance Test

Table 4 shows the change in each reach distance for the YBT between groups applying CAI.

- Anterior reach direction

In the research, the CG showed no significant change in the ANT (pre: 44.51 ± 3.51; post: 44.44 ± 3.22), with a very small effect size (*d* = −0.02 [−0.82, 0.78]). Conversely, the REG (pre: 45.18 ± 3.06; post: 55.97 ± 5.03), NG (pre: 44.86 ± 3.08; post: 53.31 ± 5.76), and RNG (pre: 46.81 ± 3.52; post: 55.23 ± 5.65) exhibited significant increases compared to before the intervention. The REG (*d* = 2.59 [1.51, 3.68]), NG (*d* = 1.83 [0.83, 2.82]), and RNG (*d* = 1.79 [0.84, 2.74]) all had very large effect sizes. For ANT, post-hoc analysis showed a significant difference between the CG and each of the three groups, and all showed significantly higher values than the CG (*p* < 0.05).

- Posteromedial reach direction

For PM, the REG (pre: 86.82 ± 6.18; post: 98.77 ± 8.22), NG (pre: 87.18 ± 8.45; post: 96.32 ± 4.84), and RNG (pre: 84.24 ± 9.86; post: 97.92 ± 7.61) showed significant increases after applying the intervention, showing a very large effect size (REG: *d* = 1.64 [0.72, 2.57]; NG: *d* = 1.33 [0.40, 2.25]; RNG: *d* = 1.55 [0.64, 2.47]). However, there was no significant change in the CG (pre: 86.42 ± 8.41; post: 85.47 ± 6.20) and the effect size was very small (*d* = −0.13 [−0.93, 0.67]).

- Posterolateral reach direction

For PL, the REG (pre: 83.90 ± 9.93; post: 94.86 ± 7.10), NG (pre: 81.75 ± 13.47; post: 94.97 ± 3.39), and RNG (pre: 79.34 ± 13.34; post: 79.34 ± 13.34) showed significant increases following the intervention with a very large effect size (REG: *d* = 1.27 [0.39, 2.15]; NG: *d* = 1.35 [0.42, 2.27]; RNG: *d* = 1.59 [0.67, 2.50]). In contrast to the other three groups, the PL of the CG remained unchanged (pre: 81.53 ± 7.04; post: 81.47 ± 6.06), with a negligible effect size that was not statistically significant (*d* = −0.01 [−0.81. 0.79]).

- Composite score

The CG showed no significant change in the CS compared to the baseline (pre: 78.69 ± 4.98; post: 78.42 ± 5.52), with a very small effect size (*d* = −0.05 [−0.85, 0.75]). However, the REG (pre: 80.96 ± 6.87; post: 93.55 ± 5.67), NG (pre: 79.40 ± 8.37; post: 90.96 ± 6.40), and RNG (pre: 79.94 ± 8.53; post: 94.60 ± 5.70) all showed significant increases after the intervention and had very large effect size (REG: *d* = 2.00 [1.02, 2.98]; NG: *d* = 1.55 [0.60, 2.50]; RNG: *d* = 2.02 [1.04, 3.00]).

#### 3.2.2. Square Hop Test

Results of hop recordings for the SHT between groups with CAI are detailed in Table 5.

In this study, no significant difference was observed in the CG compared to the baseline (pre: 25.76 ± 8.05; post: 25.99 ± 10.40), with a very small effect size (*d* = 0.02 [−0.78 to 0.82]). However, the REG (pre: 24.80 ± 8.11; post: 18.06 ± 1.86), NG (pre: 28.07 ± 10.39; post: 20.00 ± 5.17), and RNG (pre: 29.51 ± 11.49; post: 18.44 ± 3.08) showed significant increases after the intervention, and their effect sizes were very large (REG: d = −1.15 [−2.01 to −0.28]; NG: d = −0.98 [−1.87 to −0.10]; RNG: d = −1.32 [−2.20 to −0.43]).

## 4. Discussion

This study aimed to investigate the effects of rehabilitative exercise and NMES for a total of six weeks on the morphology of foot and ankle muscles and dynamic balance in individuals with CAI. The hypothesis of the current study was verified.

### 4.1. Changes in Muscle Morphology and Dynamic Balance According to Rehabilitative Exercise

The rehabilitative exercise program applied in this study was designed with exercises using a WB and balance pad. This resulted in a beneficial impact on muscle morphology and dynamic balance in individuals with CAI. Muscle strength was found to be positively correlated with morphology in a previous study [39]. In a study similar to this one, it was found that after applying HRE to the elderly for eight weeks, there was an improvement in the strength of the ankle plantar flexion and the thickness of the triceps surae [20]. The exercise was performed by bending the knees to strengthen the soleus muscle, but the gastrocnemius muscle, which is a biarticular joint muscle, was also strengthened [40]. In particular, it is suggested that the results of the study contributed to the enhancement in muscle CSA, fiber length, and pennation angle, as eccentric exercise is effective in improving these aspects [41]. In several studies, eccentric exercise has been found to improve the morphology of atrophied muscles in both patients with musculoskeletal injuries and healthy individuals [22,39,42]. The CSA of the proximal thigh region on the unilateral of a patient who underwent anterior cruciate ligament reconstruction improved after six weeks of eccentric exercise, and the muscle thickness of the triceps surae also increased when it was applied for four and eight weeks [39,42]. It is believed that the increase in fascicle length through eccentric exercise contributed to the increase in muscle CSA and thickness [42]. Eccentric exercise preferentially recruits fast-twitch fibers and overloads the muscles [43,44], thereby generating greater force and promoting muscle protein synthesis [45,46]. In other words, eccentric exercise is considered appropriate for enlarging muscle morphology based on these principles [41,47]. 

Rehabilitative exercises consisting of balance and strength training have had positive results not only on muscle morphology but also on dynamic balance. Previous studies showed the benefits of balance training that enhanced the dynamic balance in CAI [10,15,48]. Improved performance through balance training may be associated with reduced limitations of the ankle sensorimotor system in CAI [48]. In particular, training on an unstable surface increases dynamic postural control by further stimulating proprioception, an essential component of neuromuscular control [15]. Among the many unstable ground training tools, the WB is one designed to improve mechanoreceptor function and restore normal neuromuscular feedback loops [49]. Maintaining balance on a WB compared to on a stable surface or balance pad results in higher ankle frontal plane kinematics variability and muscle activity [50]. In particular, the WB causes a greater ankle inversion–eversion ROM, peak inversion velocity, and number of inversion–eversion direction changes compared to other surfaces such as the floor or a balance pad [50]. In this study, in instructing participants to touch the edge of the board to the ground as much as possible, it is speculated that it stimulated ankle muscles such as the tibialis anterior, peroneus, and gastrocnemius [49]. Although not investigated in this study, previously reported research results show that the muscle activity of the peroneal muscle is higher compared to the tibialis anterior and gastrocnemius when using a WB [51]. This might suggest that the peroneus muscles play a more active role in maintaining posture stability during WBE [51]. The principle of WBE makes them effective in reconditioning the proprioceptive system [49]. This could indicate that balance training could potentially enhance the compromised sensorimotor abilities in groups with CAI [10].

The improvement in dynamic balance observed in this study may be attributed to both strength and balance. There are reports from various studies indicating that strength training can lead to improvements in dynamic balance [52,53]. Six weeks of strength training including calf raises resulted in increased reach distances in all directions during the SEBT [53]. Furthermore, hop performance was shown to improve with strength training in various hop tests other than the square hop test [52]. Hall et al. [52] proposed that an increase in both concentric and eccentric eversion could prevent LAS by controlling the stress from eversion. That is, our knowledge could suggest that plantar flexion strength enhanced by HRE focusing on the triceps surae muscles may contribute to ankle stability during SHT. Studies have demonstrated that HRE targeted at the triceps surae can enhance plantar flexion strength in the elderly [20], patients with CAI [52], and survivors of stroke [54,55]. The increase in eccentric plantar flexion strength resulting from strength training was found to be related to improved postural control during SEBT [52]. Performing repetitively eccentric contractions provides feedback on centripetal inputs through the muscle spindle and the Golgi tendon organ, thereby promoting muscle activity [54]. In other words, it can be suggested that strength training may enhance neuromuscular and sensorimotor control, and rectify the muscle latency by boosting the function of mechanoreceptors [49]. Also, the present study utilized a modified HRE conducted on an unstable surface. This is a combined rehabilitative exercise designed to enhance strength and balance. Performing strengthening exercises on an unstable surface positively impacts the enhancement in neuromuscular control ability. A study with similar findings demonstrated that a six-week program of combined strength and balance training exercises led to improvements in ankle proprioception and both static and dynamic balance in the CAI group, compared to their pre-training state [12]. Hence, the combined rehabilitative exercise program in this study, which combines strength and balance training, may have had a positive impact on functional performance. This is likely due to the synergistic effect it created, enhancing both static and dynamic balance as well as postural control. In light of the kinetic chains, enhanced neuromuscular control in the ankle joint could influence the muscles of proximal joints like the knee and hip [36]. This heightened neuromuscular control ability in the ankle joint might have played a role in bettering YBT and SHT performance by impacting the knee and hip joints, essentially affecting the entire lower extremities through the kinetic chain [36].

### 4.2. Changes in Muscle Morphology and Dynamic Balance According to NMES

Over a period of several weeks, the use of NMES can counteract the detrimental effects of atrophy by inducing muscle contractions [56]. This, in turn, leads to improved health and quality of life for both patients and healthy individuals [57]. Based on a previous study [58], therapeutic benefits are observed after administering NMES for 30–60 min daily for a duration of six to eight weeks. This indicates that the length of NMES application in this study was instrumental in the enhancement in muscle morphological characteristics.

Another comparable study demonstrated that after four weeks of NMES treatment, there was an increase in the CSA of the tibialis anterior and gastrocnemius muscles in children diagnosed with cerebral palsy [27]. It was reported by a researcher that NMES is employed as a preventive measure against muscle atrophy following immobilization [59]. A study indicated that NMES is employed to counteract muscle atrophy following immobilization [59]. It was observed that the group that did not receive NMES for a period of five days exhibited a significant reduction in the CSA of the quadriceps muscle by 3.5% in the immobilized limb. On the other hand, the group that was subjected to NMES for five days showed no significant alterations in the CSA of the quadriceps muscle [59]. Through the use of NMES, alterations were observed in the CSA of muscle fibers [59]. Specifically, within the group that received NMES, a significant enlargement was noted in the CSA of Type II muscle fibers compared to that of Type I fibers [59]. Muscle atrophy is predominantly attributed to a reduction in the size of Type II muscle fibers [60]. The size principle of motor unit recruitment generally states that smaller motor units are engaged first, followed by the activation of larger motor units [61]. NMES is designed to preferentially recruit Type II muscle fibers, which may play a significant role in preventing muscle atrophy [60,62]. In other words, the augmentation of Type II muscle fibers could be a contributing factor to the increase in CSA. NMES has the potential to reverse muscle atrophy and weakness caused by immobilization by promoting or preserving muscle protein synthesis. This suggests that it could play a role in inhibiting proteolysis [63]. An in vivo study has shown that NMES not only accelerates the process of muscle protein synthesis but also prevents protein degradation [59,64]. This suggests that the increase in muscle fiber composition could be attributed to electrical stimulation [25]. Therefore, it has been established that NMES is beneficial in ameliorating muscle atrophy across a broad spectrum of individuals, ranging from healthy people to patients.

Based on the findings of this study, six weeks of NMES application not only stimulated muscle growth but also enhanced functional performance in individuals with CAI. Utilized to produce muscle contractions, NMES is effective in improving mobility in individuals with reduced muscle activity [57]. Previous studies have shown that using NMES improves the function and strength of weakened muscles [24,65,66]. As previously mentioned, NMES primarily aids in augmenting muscle strength by engaging large motor units. The fundamental principle of NMES is its ability to primarily stimulate motor axons, thereby inducing muscle adaptation and resulting in muscle contraction. This treatment for eight weeks influenced improving ankle dorsiflexion strength in children with unilateral spastic cerebral palsy. As aforementioned, NMES is mainly effective in improving muscle strength by recruiting large motor units. The mechanism of NMES is its primary activation of motor axons, which instigates muscle adaptation and subsequently results in muscle contraction [67]. Muscles that are contracted repeatedly over several weeks using NMES can lead to the induction of contractile capabilities and neural adaptation [56,67]. High muscle contractility, induced by electrical stimulation, enhances the development of Type II muscle fibers [65]. Activated Type II muscle fibers produce a more substantial force due to their larger size relative to Type I muscle fibers [68]. That is, NMES serves as a suitable clinical instrument capable of effectively enhancing muscle strength by triggering muscle contractions through the engagement of large motor units [57]. Therefore, the findings of this study indicate that sustained use of NMES could lead to enhanced adaptation of the neuromuscular system and that muscles activated through NMES could generate dynamic forces during functional performance evaluations.

### 4.3. Limitations

This study does have certain limitations. First, there was a sex ratio imbalance between sub-groups of CAI. Since there may be differences in muscle morphology depending on sex, it is considered necessary to balance the sex ratio in future studies. Second, the body composition was not considered in this study. Future studies should consider this aspect, as it may affect muscle morphology. Third, this research only focused on the injured limbs of participants with CAI. Given that muscle morphology and functional performance can vary depending on the dominant limb, future studies should consider investigating the dominant limb. Fourth, individuals with CAI often exhibit defects not just in the ankle joint, but also in the knee and hip joints. Therefore, future research should explore changes in muscle morphology in the proximal joint, considering that each joint is interconnected through a kinetic chain. Fifth, the results of this study exhibit spectral bias because dynamic balance was compared only with and without CAI. Because dynamic balance deficits may also be present in individuals with conditions other than CAI, obtaining a history of other foot and ankle conditions is thought to be a key element in diagnosing CAI. Therefore, future studies should investigate additional foot and ankle conditions other than CAI that could potentially contribute to balance deficits.

## 5. Conclusions

A total of six weeks of rehabilitative exercise and NMES were found to be effective in improving muscle atrophy and functional performance in individuals with CAI, regardless of the intervention methods. This study suggests that clinicians may utilize six weeks of either or both rehabilitative exercises or/and NMES to improve muscle atrophy and functional performance in people with CAI.

## Figures and Tables

**Table 1:** Demographic characteristics of the CAI participants’ sub-group (mean ± SD).

	Sub-Group				*p* Value
	CG	REG	NG	RNG	
*n*	12	12	11	12	N/A
Sex (M/F)	8:4	6:6	6:5	6:6	N/A
Age (years)	23.08 ± 1.83	22.58 ± 1.08	22.27 ± 1.19	24.17 ± 3.83	0.22
Height (cm)	173.53 ± 8.97	169.98 ± 8.41	173.95 ± 7.40	171.05 ± 8.64	0.61
Weight (kg)	73.63 ± 16.51	68.45 ± 16.13	69.19 ± 11.93	69.98 ± 19.58	0.87
CAIT	16.42 ± 2.54	15.75 ± 6.72	15.82 ± 3.76	13.92 ± 3.60	0.55
IdFAI	22.42 ± 5.50	23.58 ± 8.59	25.91 ± 4.85	23.50 ± 6.24	0.63

CAI: chronic ankle instability; CG: control group; REG: rehabilitative exercise group; NG: neuromuscular electrical stimulation group; RNG: rehabilitation and neuromuscular electrical stimulation group; CAIT: Cumberland Ankle Instability Tool; IdFAI: Identification of Functional Ankle Instability.

**Table 2:** Rehabilitative exercise program for six weeks.

Exercise	Sessions per Week	Volume	Rest in between Trials
HRE	3 sessions	15 rep × 3 sets	30 s
WBE	3 sessions	40 sec × 3 sets	1 min

HRE: heel raise exercise; WBE: wobble board exercise.

**Table 3:** Changes in the morphology of each muscle between groups with CAI (mean ± SD).

Muscle	Morphology	Group	Baseline	Post-Intervention	Cohen’s *d* ES with 95% CI
FDL	CSA (cm^2^)	CG	0.84 ± 0.17	0.82 ± 0.18	−0.16 [−0.96, 0.64]
		REG	0.86 ± 0.10	1.21 ± 0.21	2.07 [1.08, 3.06]
		NG	0.87 ± 0.07	1.19 ± 0.15	2.67 [1.52, 3.82]
		RNG	0.86 ± 0.08	1.25 ± 0.27	2.02 [1.04, 3.00]
	Thickness (cm)	CG	0.80 ± 0.09	0.79 ± 0.09	−0.14 [−0.94, 0.66]
		REG	0.83 ± 0.07	0.99 ± 0.13	1.54 [0.63, 2.45]
		NG	0.84 ± 0.08	0.98 ± 0.13	1.21 [0.30, 2.12]
		RNG	0.81 ± 0.07	1.04 ± 0.12	2.43 [1.38, 3.48]
FHL	CSA (cm^2^)	CG	4.95 ± 0.32	4.98 ± 0.30	0.08 [−0.72, 0.88]
		REG	4.97 ± 0.28	4.97 ± 0.27	0.02 [−0.78, 0.82]
		NG	4.96 ± 0.41	4.97 ± 0.37	0.03 [−0.81, 0.87]
		RNG	4.97 ± 0.26	4.98 ± 0.27	0.04 [−0.76, 0.84]
	Thickness (cm)	CG	1.36 ± 0.08	1.37 ± 0.09	0.14 [−0.66, 0.94]
		REG	1.36 ± 0.05	1.37 ± 0.06	0.15 [−0.66, 0.95]
		NG	1.36 ± 0.06	1.37 ± 0.04	0.24 [−0.60, 1.08]
		RNG	1.36 ± 0.07	1.37 ± 0.09	0.10 [−0.70, 0.90]
GA-M	CSA (cm^2^)	CG	6.81 ± 1.13	6.80 ± 0.94	0.00 [−0.80, 0.80]
		REG	6.78 ± 0.57	7.47 ± 0.83	0.96 [0.12, 1.81]
		NG	6.77 ± 0.71	7.37 ± 0.65	0.89 [0.02, 1.77]
		RNG	6.78 ± 1.05	7.57 ± 0.76	0.87 [0.03, 1.70]
	Thickness (cm)	CG	1.65 ± 0.12	1.66 ± 0.07	0.06 [−0.74, 0.86]
		REG	1.64 ± 0.12	1.90 ± 0.17	1.83 [0.88, 2.78]
		NG	1.64 ± 0.11	1.82 ± 0.15	1.37 [0.45, 2.30]
		RNG	1.64 ± 0.23	1.96 ± 0.21	1.48 [0.58, 2.38]
GA-L	CSA (cm^2^)	CG	5.08 ± 0.59	5.11 ± 0.50	0.05 [−0.75, 0.86]
		REG	5.02 ± 0.92	6.16 ± 1.39	0.97 [0.12, 1.81]
		NG	5.06 ± 0.56	5.97 ± 0.74	1.39 [0.12, 1.81]
		RNG	5.03 ± 0.57	6.32 ± 0.45	2.50 [1.43, 3.57]
	Thickness (cm)	CG	1.35 ± 0.17	1.36 ± 0.12	0.09 [−0.71, 0.89]
		REG	1.36 ± 0.25	1.63 ± 0.24	1.12 [0.26, 1.98]
		NG	1.37 ± 0.21	1.59 ± 0.12	1.36 [0.43, 2.28]
		RNG	1.36 ± 0.11	1.66 ± 0.15	2.26 [1.24, 3.28]
SOL	CSA (cm^2^)	CG	6.06 ± 0.60	6.09 ± 0.36	0.07 [−0.74, 0.87]
		REG	5.82 ± 0.60	6.95 ± 0.56	1.94 [0.97, 2.91]
		NG	5.90 ± 0.82	6.68 ± 0.80	0.96 [0.08, 1.85]
		RNG	5.93 ± 0.42	6.98 ± 0.51	2.26 [1.23, 3.28]
	Thickness (cm)	CG	1.54 ± 0.18	1.54 ± 0.17	0.02 [−0.78, 0.82]
		REG	1.52 ± 0.14	1.80 ± 0.15	1.94 [0.97, 2.91]
		NG	1.53 ± 0.13	1.76 ± 0.16	1.59 [0.64, 2.55]
		RNG	1.53 ± 0.16	1.83 ± 0.20	1.64 [0.72, 2.57]

ES: effect size; CI: confidence interval; FDL: flexor digitorum longus; FHL: flexor hallucis longus; GA-M: gastrocnemius medial head; GA-L: gastrocnemius lateral head; SOL: soleus; CSA: cross-sectional area; thickness: muscle thickness; CG: control group; REG: rehabilitative exercise group; NG: neuromuscular electrical stimulation group; RNG: rehabilitative exercise and neuromuscular electrical stimulation group.

**Table 4:** Normalized reach distances for Y-Balance test between groups with CAI (mean ± SD).

Reach Direction	Group	Baseline	Post-Intervention	Cohen’s *d* ES with 95%CI
ANT	CG	44.51 ± 3.51	44.44 ± 3.22	−0.02 [−0.82, 0.78]
	REG	45.18 ± 3.06	55.97 ± 5.03	2.59 [1.51, 3.68]
	NG	44.86 ± 3.08	53.31 ± 5.76	1.83 [0.83, 2.82]
	RNG	46.81 ± 3.52	55.23 ± 5.65	1.79 [0.84, 2.74]
PM	CG	86.42 ± 8.41	85.47 ± 6.20	−0.13 [−0.93, 0.67]
	REG	86.82 ± 6.18	98.77 ± 8.22	1.64 [0.72, 2.57]
	NG	87.18 ± 8.45	96.32 ± 4.84	1.33 [0.40, 2.25]
	RNG	84.24 ± 9.86	97.92 ± 7.61	1.55 [0.64, 2.47]
PL	CG	81.53 ± 7.04	81.47 ± 6.06	−0.01 [−0.81. 0.79]
	REG	83.90 ± 9.93	94.86 ± 7.10	1.27 [0.39, 2.15]
	NG	81.75 ± 13.47	94.97 ± 3.39	1.35 [0.42, 2.27]
	RNG	79.34 ± 13.34	95.60 ± 5.70	1.59 [0.67, 2.50]
CS, %	CG	78.69 ± 4.98	78.42 ± 5.52	−0.05 [−0.85, 0.75]
	REG	80.96 ± 6.87	93.55 ± 5.67	2.00 [1.02, 2.98]
	NG	79.40 ± 8.37	90.96 ± 6.40	1.55 [0.60, 2.50]
	RNG	79.94 ± 8.53	94.60 ± 5.70	2.02 [1.04, 3.00]

ES: effect size; CI: confidence interval; ANT: anterior; PM: posteromedial; PL: posterolateral; CS: composite score; CG: control group; REG: rehabilitative exercise group; NG: neuromuscular electrical stimulation group; RNG: rehabilitative exercise and neuromuscular electrical stimulation group.

**Table 5:** Records for square hop test between groups with CAI (mean ± SD).

Variable	Group	Baseline	Post-Intervention	Cohen’s *d* ES with 95% CI
Square hop test (s)	CG	25.76 ± 8.05	25.99 ± 10.40	0.02 [−0.78, 0.82]
	REG	24.80 ± 8.11	18.06 ± 1.86	−1.15 [−2.01, −0.28]
	NG	28.07 ± 10.39	20.00 ± 5.17	−0.98 [−1.87, −0.10]
	RNG	29.51 ± 11.49	18.44 ± 3.08	−1.32 [−2.20, −0.43]

ES: effect size; CI: confidence interval; CG: control group; REG: rehabilitative exercise group; NG: neuromuscular electrical stimulation group; RNG: rehabilitative exercise and neuromuscular electrical stimulation group.

## Data Availability

The data presented in this study are available on request from the corresponding author.
